# Disentangling Structural and Electronic Contributions to Photogenerated Mobile Charge Carrier Yield and Transport in Fe_2_O_3_ Polymorphs

**DOI:** 10.1021/acsami.5c23252

**Published:** 2026-02-19

**Authors:** Sa’ar Shor Peled, Kumaraswamy Miriyala, Daniel A. Grave

**Affiliations:** † Department of Materials Engineering, 26732 Ben-Gurion University of the Negev, Beer Sheva 8410500, Israel; ‡ Ilse Katz Institute for Nanoscale Science and Technology, Ben-Gurion University of the Negev, Beer Sheva 8410500, Israel

**Keywords:** α-Fe_2_O_3_, β-Fe_2_O_3_, photogeneration yield, spatial collection efficiency, photoelectrochemical water-splitting

## Abstract

Iron oxides exhibit poor photoconversion efficiencies as photoelectrodes for solar water splitting, generally attributed to short carrier diffusion lengths and subunity yields of photogenerated mobile charge carriers caused by ultrafast relaxation through ligand field (LF) states. However, the extent to which crystal structure or electronic configuration governs these loss mechanisms remains unclear. Here, epitaxial thin-film photoanodes of the α- and β-Fe_2_O_3_ polymorphs, which share the same Fe^3+^ (3d^5^) electronic configuration yet possess distinct crystal symmetries, are employed as model systems to disentangle the relative influence of electronic configuration and crystal structure on charge carrier yields and transport. Using a computational method that combines optical and photoelectrochemical measurements, we determine both the wavelength-dependent efficiency of mobile charge carrier generation and the depth-dependent charge collection probability. We find that structural factors influence charge carrier transport, with the α-Fe_2_O_3_ films exhibiting a larger hole transport length than the *β*-Fe_2_O_3_ thin films. In contrast, both polymorphs show an essentially identical spectral profile for mobile-carrier generation, indicating that the energies of the ligand-to-metal charge-transfer (LMCT) transitions that produce mobile carriers are largely unaffected by the difference in crystal structure. These results suggest that carrier yields are governed predominantly by the local Fe–O electronic structure associated with the octahedrally coordinated Fe^3+^ centers and are consistent with the view that ligand-field states act as intrinsic nonproductive relaxation pathways in open d-shell metal oxides.

## Graphical abstract

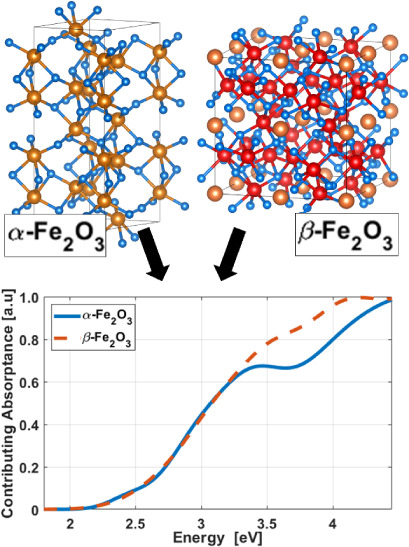

## Introduction

Photoelectrochemical (PEC) water splitting has been extensively studied as a promising pathway for producing solar-driven green hydrogen.^1,2^ Due to its suitable bandgap energy (∼2.1 eV), nontoxicity, stability in aqueous solution, and earth-abundance, hematite (α-Fe_2_O_3_) is one of the most thoroughly studied photoelectrode materials.^3^ However, even the most efficient α-Fe_2_O_3_ photoanodes achieve about half the theoretical current density limit of 12.6 mA cm^–2^ predicted under standard sunlight illumination (AM1.5G).^4–6^ These losses are primarily attributed to short minority carrier diffusion lengths, reported to be between 2 and 15 nm,^7,8^ and more recently to subunity yields of mobile charge carriers following photoexcitation, where only a fraction of absorbed photons result in the generation of mobile carriers capable of contributing to photocurrent.^6,9–11^ Efforts to mitigate the first limitation have primarily involved approaches such as nanostructuring or resonant light trapping that can bridge the mismatch between optical thickness required for sufficient light absorption and poor charge carrier transport.^3,12,13^ Additionally, doping with aliovalent cations such as Ti^4+^ has been shown to reduce recombination and improve charge carrier collection,^7,14,15^ while surface modification via bandgap engineering and applying cocatalytic overlayers such as Co–Pi or Fe_(1–*x*)_Ni_*x*_OOH has also proven effective in enhancing interfacial charge transfer kinetics and lowering the onset potential for oxygen evolution.^6,8,16–20^

Although similar approaches have enabled near-ideal performance in closed- and empty-d-shell materials such as BiVO_2_ and TiO_2_,^21,22^ they have proven far less effective for hematite and other open d-shell oxides, where subunity mobile charge carrier yields have been suggested to fundamentally limit performance. Indeed, early studies of hematite photoanodes noted a significant discrepancy between the incident photon conversion efficiency (IPCE) spectrum and absorption spectrum.^23,24^ In recent years, several explanations have been suggested to account for this loss mechanism, including optical absorption through ligand field (LF) transitions which do not contribute to mobile charge carrier generation, but rather produce excited states that are site localized.^11^ Other potential explanations include excitation-wavelength-dependent small polaron trapping following optical absorption into charge transfer states,^10^ as well as direct optical excitation into polaronic states from the ground state.^25^ Recent work has shown that the existence of LF states in open d-shell metal-oxides results in ultrafast relaxation pathways that reduce carrier yields relative to metal-oxides with d^0^ or d^10^ electronic configuration.^9^

While the microscopic origin of the ultrafast carrier losses in hematite remains under debate, these losses result in a wavelength-dependent photogeneration yield, ξ_(λ)_, that reflects variations in mobile charge carrier generation efficiency as a function of excitation energy.^6^ Quantifying ξ_(λ)_ provides insight into the factors governing these losses and offers pathways to improve mobile charge-carrier yields in transition-metal oxides. In previous works, combined experimental and computational approaches were applied to α-Fe_2_O_3_, using IPCE spectra, optical measurements and modeling, and photoelectrochemical (PEC) characterization within an algorithmic framework to extract both ξ_(λ)_ and the depth-resolved spatial charge collection efficiency, ϕ_(*x*)_.^16,26^ Complementary time-resolved microwave conductivity (TRMC) measurements showed that the spectral profile of the TRMC quantum yield-mobility product on nanosecond time scales followed the same wavelength dependence as the ξ_(λ)_ spectrum extracted from ϕ_(*x*)_ analysis.^27^ Together, these analyses showed that only a fraction of the absorption led to charge carrier photogeneration and transport on device-relevant time scales, and that the mobile charge carrier yield exhibits a distinct wavelength dependence, decreasing sharply toward the band edge. Despite these advancements, it is unclear what material characteristics fundamentally govern ξ_(λ)_ losses.

To address this question, this work employs model epitaxial thin film photoanodes of two Fe_2_O_3_ polymorphs, α- and β-Fe_2_O_3_, to disentangle structural and electronic contributions to carrier photogeneration and transport. The use of epitaxial films minimizes the influence of randomly oriented grains and microstructural disorder, thereby reducing optical losses associated with scattering and reflection and enabling a more direct assessment of intrinsic carrier generation and transport properties. The metastable β-Fe_2_O_3_ polymorph serves as a structural counterpart to α-Fe_2_O_3_, as both share the same chemical composition and 3d^5^ electronic configuration of the Fe^3+^ cations but possess different crystal structures and local bonding environments. β-Fe_2_O_3_ adopts a cubic bixbyite structure containing two inequivalent octahedral Fe^3+^ sites, which contrasts with the corundum lattice of α-Fe_2_O_3_ where all the octahedral Fe^3+^ sites are equivalent.^28^ Although metastable under ambient conditions, β-Fe_2_O_3_ photoanodes have been synthesized by chemical solution routes^29–32^ and can also be stabilized epitaxially using techniques such as atomic layer deposition^33,34^ or, by pulsed laser deposition (PLD) as recently demonstrated by our group and others.^35,36^ While β-Fe_2_O_3_ exhibits a narrower optical bandgap than α-Fe_2_O_3_ which is attractive for solar water splitting, its charge-transport and photogeneration characteristics remain largely unexplored. To probe how these structural differences influence carrier generation and transport, we extract the wavelength-dependent photogeneration yield ξ_(λ)_ and the depth-resolved spatial charge collection efficiency ϕ_(*x*)_ for both polymorphs. While structural factors significantly influence charge-carrier transport, the wavelength-dependent spectral profile of the absorption that contributes to mobile carrier generation is nearly identical for the two polymorphs. This indicates that the spectral dependence of mobile carrier generation is governed primarily by the Fe^3+^ (3d^5^) electronic configuration, whereas structural variations mainly affect charge-carrier transport.

## Experimental Section

### Device Fabrication

Metal-oxide thin film deposition was carried out by pulsed laser deposition. Commercial Nb-doped SnO_2_ (NTO, PLD Targets, 0.66%at Nb, 2 in.) and indium tin-oxide (ITO, Kurt J. Lesker, 90/10%, 2 in.) targets were used for deposition of the transparent conducting oxide (TCO) layers. A homemade α-Fe_2_O_3_ target was synthesized using a α-Fe_2_O_3_ (Acros Organics, 99.999%) powder that was ball-milled and sintered at 1200 °C for 8 h. Single crystal c-plane sapphire (Al_2_O_3_(0001), Cryscore) and yttrium stabilized zirconia (YSZ(111), Maideli Advanced Material CO., LTD) substrates were used for the growth of α- and β-Fe_2_O_3_, respectively. The PLD chamber was evacuated to <5 × 10^–6^ Torr base pressure and substrate set temperatures were elevated to 800 or 500 °C for α- and β-Fe_2_O_3_ depositions, respectively. Subsequently, the chamber was pressurized with oxygen to 5 mTorr or 10 mTorr (for TCO and Fe_2_O_3_ depositions, respectively). Targets were preablated with a closed shutter. A KrF excimer laser (Coherent Compex 102F, 248 nm) was used to ablate the targets with a fluence of approximately 1 J cm^–2^ and with a repetition rate of 3 Hz.

### Characterization

High-resolution X-ray diffraction (HRXRD) studies were performed to examine the crystallinity, phase purity, and orientation of the films, using a Panalytical Empyrean III X-ray diffractometer (Cu K_α_ radiation) accompanied by a 2-bounce monochromator (Ge (220) channel-cut) which was operated at 1.8 kW. Prior to the measurements, precise alignment steps were performed to adjust the height of the sample. High-resolution θ – 2θ scans were performed to identify the out-of-plane crystallographic orientation of the TCO and Fe_2_O_3_ films. Peaks were assigned with ICDD PDF-4 #01-086-8560 (NTO), #01-083-3352 (ITO), #00-033-0664 (α-Fe_2_O_3_) and #01-083-8470 (β-Fe_2_O_3_).

UV–vis spectrophotometry (SP) was performed using an Agilent CARY5000 system with universal measurement accessory (UMA), scanning from 1000 to 200 nm. Spectroscopic ellipsometry (SE) measurements were carried out using a J.A. Woollam M-RC2 ellipsometer measuring at 55–75° angle of incidence in the wavelength range of 300 to 1200 nm and analyzed using J.A. Woollam CompleteEase v.6.7 software.

Mott–Schottky (MS) and intensity-modulated photocurrent spectroscopy (IMPS) measurements were used to calculate the depletion width and charge transfer efficiency, respectively. MS and IMPS measurements were performed using a Zennium pro electrochemical workstation (Zahner) equipped with a white LED light source outputting an intensity of 100 mW cm^–2^. For IMPS, a sinusoidal modulated light intensity was applied with a modulation depth of 15% in the frequency range of 10 kHz to 300 mHz. Linear-sweep voltammetry (LSV) measurements were performed using Palmsens 4 potentiostat and ScienceTech solar simulator system under AM1.5G illumination. Measurements were performed in a three-electrode configuration with a platinum counter electrode and Hg/HgO reference electrode. Incident photon-to-current conversion efficiency (IPCE) measurements were performed using ScienceTech PTS-2 system with a spectral range of 290 to 650 nm with a step size of 2 nm, and with an aperture size of 0.1075 cm^–2^.

## Results and Discussion

### Structural Analysis

Epitaxial α-Fe_2_O_3_ and β-Fe_2_O_3_ thin-film photoanodes with a thickness of 20 nm were grown by pulsed laser deposition on Al_2_O_3_(0001) and YSZ(111) single crystal substrates coated with epitaxial transparent conducting oxide (TCO) layers of Nb-doped SnO_2_ (NTO) and indium-doped tin oxide (ITO), respectively. High-resolution X-ray diffraction (HRXRD) θ-2θ patterns are presented in Figures S1a and S1b, where a single out-of-plane orientation is confirmed for both the TCO and Fe_2_O_3_ as follows: α-Al_2_O_3_(0001)/NTO­(100)/α-Fe_2_O_3_(0001) and YSZ(111)/ITO(111)/β-Fe_2_O_3_(111). High-resolution rocking curve scans for the α-Fe_2_O_3_ (0006) and β-Fe_2_O_3_ (222) reflections (Figures S1c and S1d) show full-width-half-maximum values of 0.1° and 0.4°, respectively, demonstrating that the films possess small out-of-plane mosaic spread. Physical vapor deposition techniques such as PLD are known to produce high-quality films,^37,38^ as evidenced here by the high crystalline quality observed in XRD measurements and by ultrasmooth surfaces confirmed through AFM analysis (Figures S1e and S1f), showing subnanometer (<1 nm) surface roughness. The low roughness is important to mitigate light scattering that may interfere with the optical measurements and simulations, and surface effects that can cause unwanted complexities during the spatial collection efficiency analysis. Further structural characterization of representative films can be found in our previous works.^36,39^

### Spatial Collection Efficiency Analysis

The relationship between ξ_(λ)_, ϕ_(*x*)_, and the measured spectral response can be expressed by eq 1, which describes the mathematical representation of the IPCE spectrum of a photoactive device according to the following integral:

1 $$IPCE_{\left(\lambda \right)}=\int_{0}^{d}\frac{I_{\left(\lambda ,x\right)}}{I_{0_{\left(\lambda \right)}}}\alpha_{\left(\lambda \right)}\xi_{\left(\lambda \right)}\phi_{\left(x\right)}dx$$

where *d* is the device thickness, *x* is the depth from the front surface, $\frac{I_{\left(\lambda ,x\right)}}{I_{0_{\left(\lambda \right)}}}$ is the wavelength-dependent photon flux at distance *x* from the surface normalized by the incident flux at the surface, and α_(λ)_ is the absorption coefficient. The product $\frac{I_{\left(\lambda ,x\right)}}{I_{0_{\left(\lambda \right)}}}\alpha_{\left(\lambda \right)}$ is the optical generation efficiency (OG_(*x*,λ)_) within the photoactive layer.

As evident from eq 1, the ξ_(λ)_ and ϕ_(*x*)_ terms are coupled and cannot be independently determined from conventional IPCE measurements. In addition, eq 1 represents a Fredholm integral equation of the first kind. Such equations are ill-posed inverse problems that may have an infinite number of possible solutions. Computational approaches based on numerical techniques have been introduced to identify physically consistent solutions,^16,26,40–42^ typically by constraining the magnitude of ξ_(λ)_ or ϕ_(*x*)_ based on a priori assumptions.

In this work, we employ the algorithmic process presented in Figure 1 to solve eq 1 and extract the ϕ_(*x*)_ and ξ_(λ)_ quantities for both polymorphs. The first inputs into the algorithm are the experimentally measured IPCE spectra under three operating potentials (1.43, 1.53, and 1.63 *V*_RHE_, chosen based on linear sweep voltammetry (LSV) measurements presented in the PEC characterization section). For each potential, multiple IPCE measurements were made and the associated error is shown in Figure S4. The second input is the wavelength-depth dependent optical generation efficiency, OG_(*x*,λ)_, which was obtained from transfer matrix method simulations following extraction of the optical constants through spectroscopic ellipsometry measurements and validated against spectrophotometry measurements. Detailed optical analysis of the two polymorphs will be presented in the next section.

**Figure 1:**
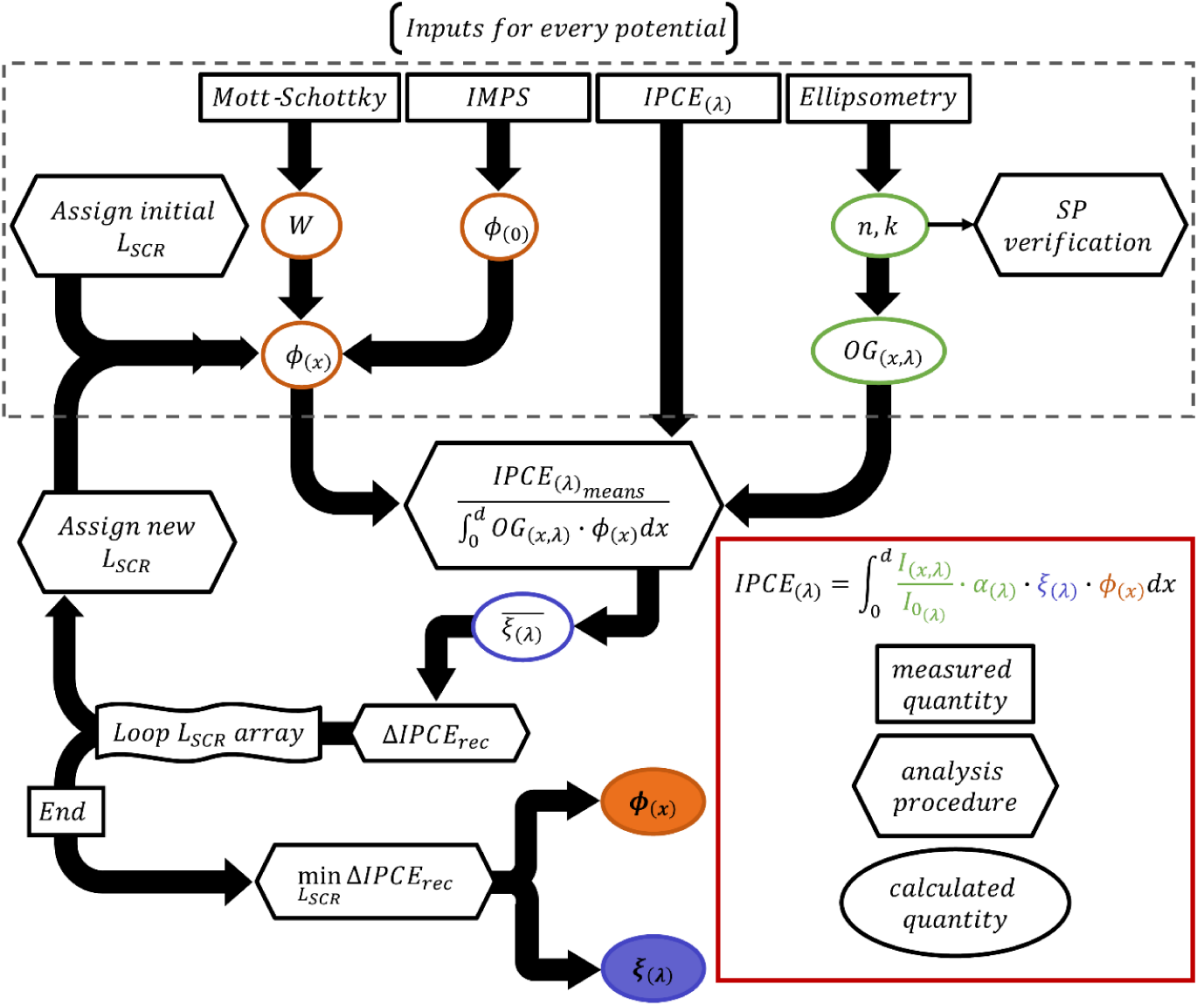
Schematic workflow of the algorithm. The legend (bottom right) indicates the meaning of each color and shape in the diagram (rectangles, trapezoids, ovals). The procedure enclosed by the dashed box was performed concurrently for all data sets (IPCE spectra measured at different potentials), and the averaged photogeneration yield, $\overset{―}{\xi_{\left(\lambda \right)}\;}$ , was obtained by averaging the resulting *ξ*_(*λ*)_ from each data set.

ϕ_(*x*)_ was parametrized according to classical semiconductor transport theory using the piecewise function shown in eq 2,^26^ which includes two characteristic transport lengths associated with the space-charge region (*L*_SCR_) and quasi-neutral region (*L*_QNR_):

2 $$\left[\begin{array}{lll} x<W & \to & \phi_{\left(x\right)}=\phi_{\left(0\right)}\exp\left(-\frac{x}{L_{SCR}}\right)\\ x>W & \to & \phi_{\left(x\right)}=\phi_{\left(0\right)}\exp\left(-\frac{W}{L_{SCR}}\right)\exp\left(-\frac{x-W}{L_{QNR}}\right) \end{array}\right]$$

Additional physical parameters were experimentally determined to constrain the ϕ_(*x*)_ solution. These include the transfer efficiency (ϕ_(0)_, also known as the charge collection probability at the surface) which was extracted from intensity-modulated photocurrent spectroscopy (IMPS) measurements, and the width of the space charge region (*W*), extracted from Mott–Schottky analysis of capacitance–potential measurements.

ϕ_(0)_ was calculated from IMPS spectra according to eq 3:^43^

3 $$\phi_{\left(0\right)}=\frac{\mathrm{LFI}}{\mathrm{HFI}}$$

where LFI and HFI are the intercepts of the low- and high-frequency semicircles of the IMPS measurement, respectively, as presented in Figure S5. Mott–Schottky analysis (Figure S6) showed that the films are fully depleted at all operating potentials used during the IPCE measurements, so only the first part (*x* < *W*) of eq 2 with a single characteristic transport length was considered when assigning the ϕ_(*x*)_ profile. Following the assignment of the ϕ_(*x*)_ profiles, ξ_(λ)_ spectra were calculated for every applied potential using eq 4:

4 $$\xi_{\left(\lambda \right)}=\frac{IPCE_{\left(\lambda \right)}}{\int_{0}^{d}OG_{\left(x,\lambda \right)}\phi_{\left(x\right)}dx}$$

The ξ_(λ)_ spectra obtained from eq 4 for each potential were averaged to one value, $\left(\overset{―}{\xi_{\left(\lambda \right)}\;}\right)$ , and used to reconstruct the measured IPCE spectra. For each potential, a broad range of transport lengths was iteratively tested, and the optimal values were determined by minimizing the root-mean-square (RMS) error between the reconstructed and experimental IPCE spectra across all three bias conditions simultaneously.

### Optical Analysis and Photoelectrochemical Performance

To calculate the optical generation efficiencies of the two polymorphs, first the complex optical constants (refractive index, *n*, and extinction coefficient, *k*) were determined by spectroscopic ellipsometry (SE) and are presented in Figure 2a and Figure 2b. The corresponding ellipsometry fitting (Figures S2 and S3) demonstrates good agreement between the measured and modeled spectra. The fitted model parameters are listed in Table S1. Significant differences are observed between the two phases. The optical constants of α-Fe_2_O_3_ show several characteristic features at wavelengths of 300–600 nm, consistent with earlier reports.^26,44^ In comparison, β-Fe_2_O_3_ shows a nearly featureless extinction coefficient while the refractive index also shows fewer features, with a broad peak around 490 nm. Additionally, the extinction coefficient of α-Fe_2_O_3_ is larger than that of the β phase, suggesting that more light can be absorbed in thinner films of α-Fe_2_O_3_, a distinct advantage given the poor charge transport properties in iron-oxide based materials where smaller thicknesses are preferred to reduce recombination. To our knowledge, spectroscopic ellipsometry of β-Fe_2_O_3_ has not been reported previously in the literature, but the near featureless optical constants we observe and the smaller band edge (∼1.8 eV) are consistent with our absorptance spectra measured by UV–vis spectrophotometry and also with earlier spectrophotometry-based reports by several authors.^29–34,36^

**Figure 2:**
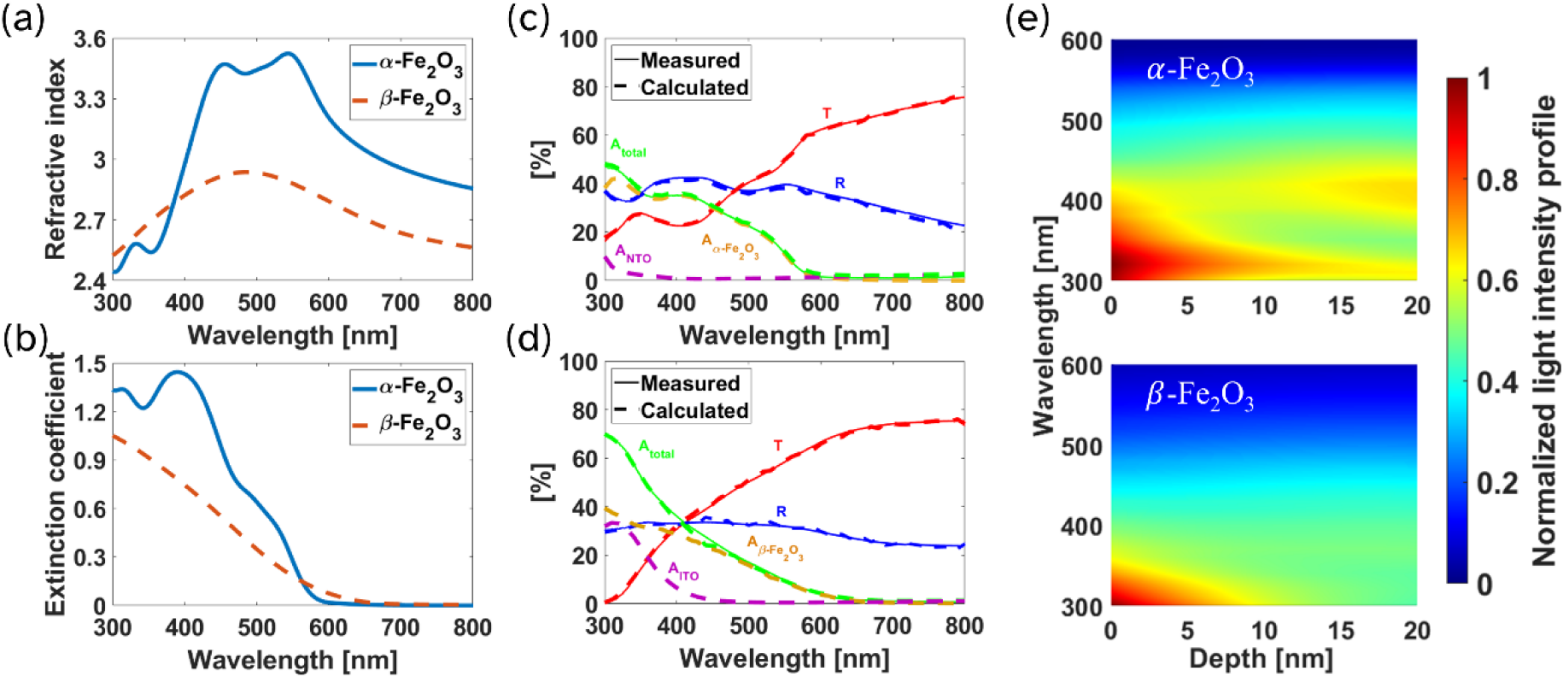
Optical and PEC performance of α- and β-Fe_2_O_3_ films. (a) Refractive indices and (b) extinction coefficients, as extracted from spectroscopic ellipsometry measurements. Simulated and measured transmittance, reflectance, and absorptance spectra of (c) α-Fe_2_O_3_ and (d) β-Fe_2_O_3_ full stacks, showing the absorption in the iron oxide layers, as well as in the transparent conductive oxide beneath. (e) Wavelength-depth resolved absorbed light intensity maps, showing clearly the difference in absorption distribution in (top) α- and (bottom) β-Fe_2_O_3_.

To validate the extracted optical constants and evaluate the optical response of the complete photoanode stack, transfer-matrix-method (TMM) simulations were performed to calculate specular reflectance (*R*), transmittance (*T*), and absorptance (*A* [%] = 100 – *R* – *T*) spectra. The simulated spectra show excellent agreement with measured UV–Vis data (Figure 2c and Figure 2d), confirming the accuracy of the optical modeling. In addition to the total absorptance (*A*_tot_) of the full stack, Figure 2c and Figure 2d show the individual layer contributions, including parasitic absorption in the current collectors (*A*_ITO_ and *A*_NTO_) and absorption within the Fe_2_O_3_ photoabsorber layers. The ITO current collector shows higher parasitic absorptance than NTO, absorbing photons with wavelengths up to ∼450 nm. Two factors can account for this effect: (i) the lower extinction coefficient of β phase relative to α (Figure 2b) which results in reduced absorption within the photoabsorber layer, and (ii) the smaller bandgap of ITO (∼3.2 eV) relative to NTO (∼3.5 eV) which extends its absorptance to longer wavelengths. Comparing the optical generation (OG) maps of the devices (Figure 2e), it is clear that α-Fe_2_O_3_ absorbs more strongly over most of the visible spectrum relative to β-Fe_2_O_3_.

LSV measurements of the two polymorphs are presented in Figure 3a, and reveal that α-Fe_2_O_3_ outperform β-Fe_2_O_3_ throughout the potential range, with lower onset potential and higher photocurrent density. To correlate the optical response with photoelectrochemical performance, the IPCE spectra of both photoanodes were measured (Figure 3b). Comparison of the absorptance (Figure 3c) and IPCE spectra of the two phases reveals clear differences in both magnitude and spectral shape. However, the absorbed photon-to-current conversion efficiency (APCE, Figure 3d), obtained by dividing the IPCE by the absorptance of the Fe_2_O_3_ absorber, exhibits nearly identical spectral behavior and magnitude for both polymorphs between 400 and 500 nm. Within this range, α-Fe_2_O_3_ shows higher total absorptance, yet this does not translate into a higher APCE. The OG maps (Figure 2e) reveal that, due to interference effects between the α-Fe_2_O_3_ and NTO layers, these additional photons are absorbed mostly near the back contact. The combination of higher absorption but unchanged APCE therefore implies that either (a) these excitations generate immobile or rapidly localized charge carriers (low ξ_(λ)_), or (b) mobile carriers generated near the back interface experience poor collection due to short transport lengths and enhanced recombination (low ϕ_(*x*)_). The decoupling analysis presented below distinguishes between these two loss mechanisms, using IPCE measurements taken under 1.43, 1.53, and 1.63 V_RHE_, where, as can be seen in the LSV measurements (Figure 3a) the devices produce sufficient photocurrent, and the dark current remains negligible.

**Figure 3:**
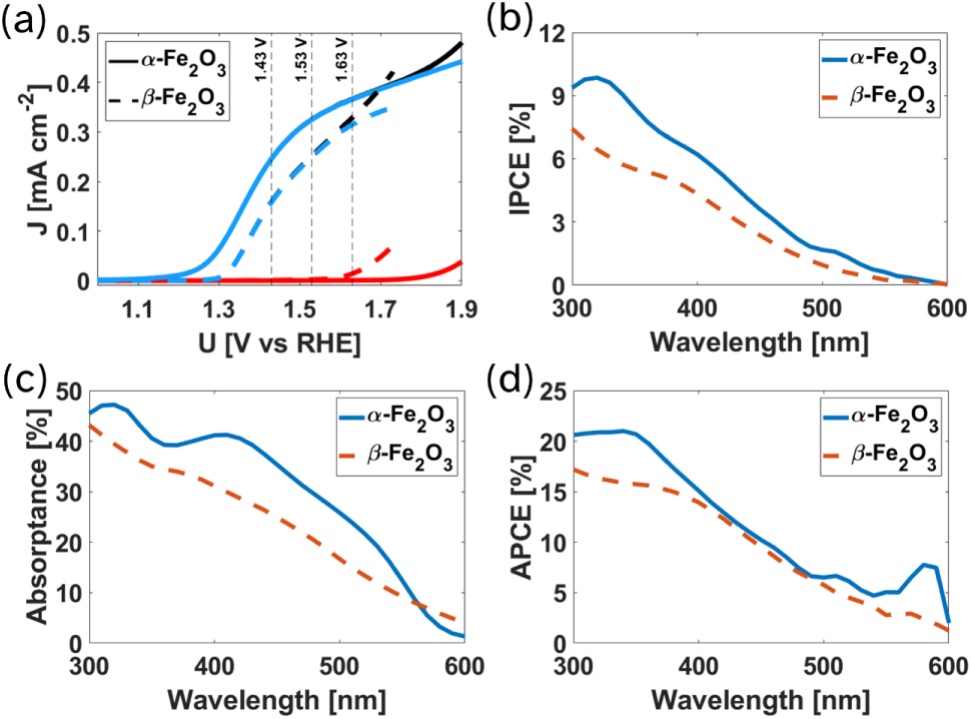
PEC performance comparison. (a) Linear sweep voltammetry measurements of α-Fe_2_O_3_ (solid lines) and β-Fe_2_O_3_ (dashed lines), showing light, dark and photocurrent densities (black, red and blue, respectively), with vertical lines mark the operating potentials chosen for IPCE measurements. (b) Incident photon-to-current conversion efficiency (IPCE) of both polymorphs measured at 1.43 V_RHE_ in 1 M NaOH electrolyte, (c) absorptance comparison of only the absorber layers without parasitic absorption and (d) absorbed photon-to-current conversion efficiency (APCE) spectra.

### Extraction ϕ_(*x*)_ of and ξ_(λ)_

The low IPCE and APCE values indicate significant losses during the photoconversion process. However, these measurements alone cannot distinguish whether the losses arise from inefficient charge carrier collection or from subunity yields of mobile charge carriers. To decouple these effects, the spatial collection efficiency analysis shown in Figure 1 was applied, combining the optical modeling and photoelectrochemical data to extract ξ_(λ)_ and ϕ_(*x*)_ for both polymorphs.

The resulting ϕ_(*x*)_ profiles are shown in Figure 4a, and the corresponding values of *L*_scr_ and ϕ_(0)_ are summarized in Table 1. ϕ_(*x*)_ is consistently higher for α-Fe_2_O_3_ than for β-Fe_2_O_3_ throughout the film depth and at all applied potentials. Quantitatively, the characteristic hole-transport length *L*_scr_ of α-Fe_2_O_3_ increases from 10.4 nm at 1.43 *V*_RHE_ to 11.8 nm at 1.63 *V*_RHE_, whereas β-Fe_2_O_3_ shows shorter transport lengths of 6.4–7.6 nm. Thus, the epitaxial hematite films exhibit a roughly 60% longer hole-transport length on average. The surface charge-transfer probability, ϕ_(0)_, extracted from IMPS measurements is similar for both polymorphs (≈0.9–1.0), indicating that the observed differences in ϕ_(*x*)_ primarily arise from bulk transport rather than surface effects. For both devices, the decrease in ϕ(0) between 1.43 and 1.53 V_RHE_ is consistent with enhanced surface recombination at lower bias, as evidenced by the larger diameter of the high-frequency semicircle in the IMPS Nyquist plots at lower operating potentials, which reflects increased recombination current. This interpretation is further supported by the LSV measurements (Figure 3a), where at 1.43 *V*_RHE_ the devices are still within the initial photocurrent rise, whereas at 1.53 *V*_RHE_ they operate in the photocurrent plateau region where surface recombination is minimized.^45^

**Figure 4:**
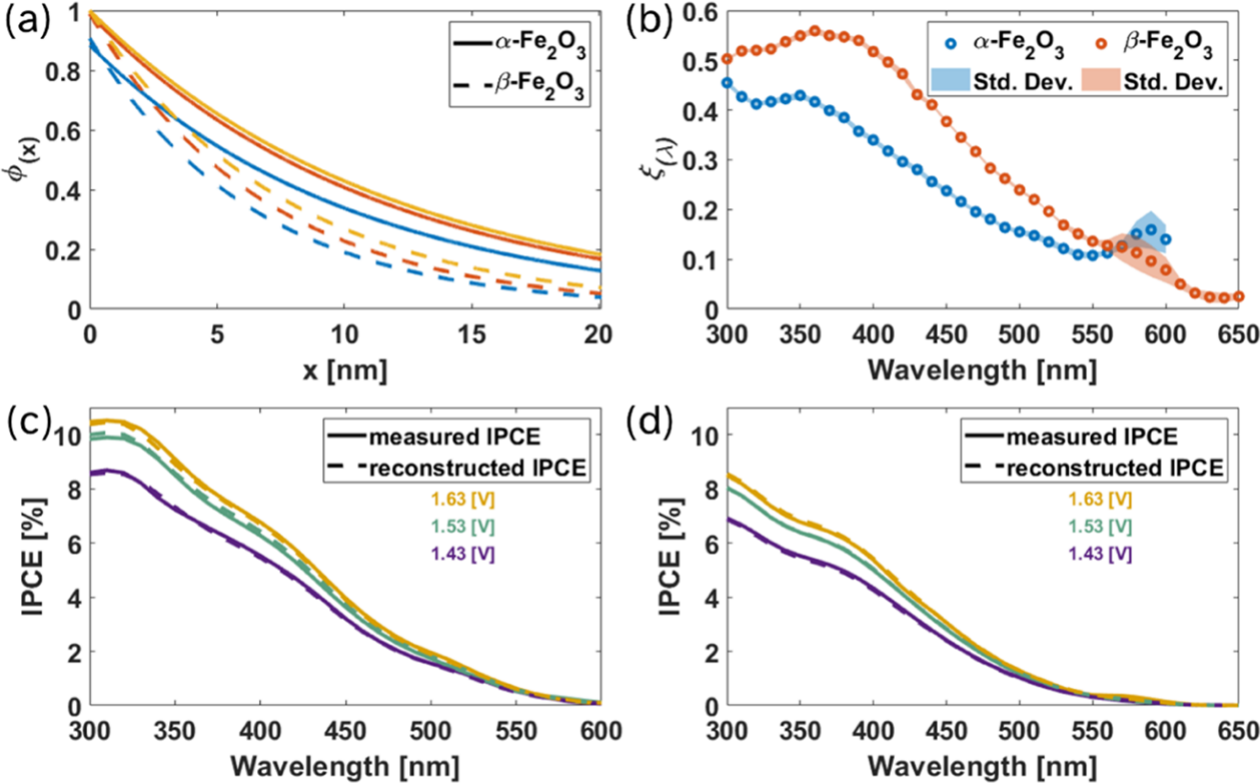
(a) ϕ_(*x*)_ profile of α-Fe_2_O_3_ (solid) and β-Fe_2_O_3_ (dashed) under applied bias of 1.43, 1.53, and 1.63 V_RHE_ (blue, red and orange curves, respectively). (b) ξ_(λ)_ spectra of α-Fe_2_O_3_ (blue) and β-Fe_2_O_3_ (red), with the shaded region representing the RMS error between the ξ_(λ)_ values extracted for each potential. Measured and reconstructed IPCE spectra of (c) α-Fe_2_O_3_ and (d) β-Fe_2_O_3_.

**Table 1:** Summary of Transport Quantities Extracted from Spatial Collection Efficiency Analysis^a^

	α-Fe_2_O_3_		β-Fe_2_O_3_	
V_RHE_ [V]	ϕ_(*0*)_	*L*_SCR_ [nm]	ϕ_(*0*)_	*L*_SCR_ [nm]
1.43	0.88	10.4 ± 0.1	0.91	6.4 ± 0.1
1.53	0.99	11.3 ± 0.1	0.99	6.8 ± 0.1
1.63	1	11.8 ± 0.1	1	7.6 ± 0.1

a Error in *L*_SCR_ was considered as the step size in the *L*_SCR_ survey.

While α-Fe_2_O_3_ exhibits a larger hole-transport length in these epitaxial films, we note that the extracted transport lengths may also reflect other polymorph-dependent structural factors, such as epitaxial quality and defect density. Recent ab initio calculations on the β-Fe_2_O_3_ phase have been reported,^46^ providing the first detailed computational study of its electronic band structure. However, to our knowledge, quantitative parameters relevant to transport properties such as carrier effective masses or mobilities have not yet been evaluated for this polymorph. Our analysis thus provides an experimental pathway to probe these transport differences through the ϕ_(*x*)_ profiles.

To further disentangle transport from carrier-generation effects, we next examine ξ_(λ)_. Although β-Fe_2_O_3_ exhibits a shorter hole-transport length than α-Fe_2_O_3_, it shows a slightly higher ξ_(λ)_ throughout most of the measured spectrum (Figure 4b). In both polymorphs, ξ_(λ)_ decreases monotonically toward longer wavelengths, indicating that higher-energy photon absorption results in a larger fraction of mobile charge carriers. The onset of this decrease occurs at longer wavelengths for β-Fe_2_O_3_, consistent with a redshift relative to α-Fe_2_O_3_. Verification of the PGY spectra was carried out by additional analysis of a set of thicker polymorph films, and is presented in Figure S7. In the case of thicker films, the depletion was only partial and the full form of eq 2 was applied. Nonetheless, the PGY spectra are in agreement with those calculated for the 20 nm films. To gain a clearer understanding of how this wavelength-dependent behavior influences the overall photoconversion efficiency, the relationship between ξ_(λ)_ and the material’s optical absorption is examined in the next section. Specifically, the total absorption coefficient is decomposed into contributing and noncontributing components to distinguish optical transitions that generate mobile carriers from those leading to localized excited states.

### Contributing and Non-Contributing Optical Absorption

To identify which optical transitions contribute to mobile charge carrier generation, the total absorption coefficient, α_tot_, was decomposed into contributing and noncontributing components according to eq 5:

5 $$\begin{array}{l} \alpha_{tot}=\frac{4\pi k}{\lambda }\\ \alpha_{C}=\alpha_{tot}\xi_{\left(\lambda \right)}\\ \alpha_{NC}=\alpha_{tot}\left(1-\xi_{\left(\lambda \right)}\right) \end{array}$$

where *k* is the extinction coefficient extracted through SE measurements, λ is the wavelength and ξ_(*λ*)_ is the previously defined photogeneration yield spectrum. The contributing absorption coefficient, *α*_C_, represents the portion of optical absorption that gives rise to mobile charge carriers, while α_NC_ corresponds to noncontributing absorption associated with localized or nonmobile excitations. Comparing these quantities provides direct insight into how efficiently absorbed photons generate mobile carriers in each polymorph, independent of transport limitations.

Figure 5a and Figure 5b present α_tot_, α_C_, and α_NC_ for α-Fe_2_O_3_ and β-Fe_2_O_3_, respectively. The shaded areas illustrate the fraction of absorption that produces mobile charge carriers versus localized excitations. In both materials, only a small fraction of the total optical absorption gives rise to mobile charge carriers at longer wavelengths, as indicated by the relatively low magnitude of α_C_ compared to α_tot_ near the band edge. The α_C_ spectra of the two polymorphs are similar in both shape and magnitude (compared directly in Figure S8), with α-Fe_2_O_3_ exhibiting a slightly higher value across most of the visible range. This indicates that although α-Fe_2_O_3_ has a smaller absolute photogeneration yield (Figure 4b), its effective carrier generation for a given film thickness is greater because of its larger absorption coefficient. In contrast, α_NC_ differs substantially: hematite displays a much larger and more spectrally structured α_NC_, whereas β-Fe_2_O_3_ shows a smoother and lower-magnitude profile. This indicates that much of the additional UV–visible absorption in α-Fe_2_O_3_ does not contribute to mobile carrier generation.

**Figure 5:**
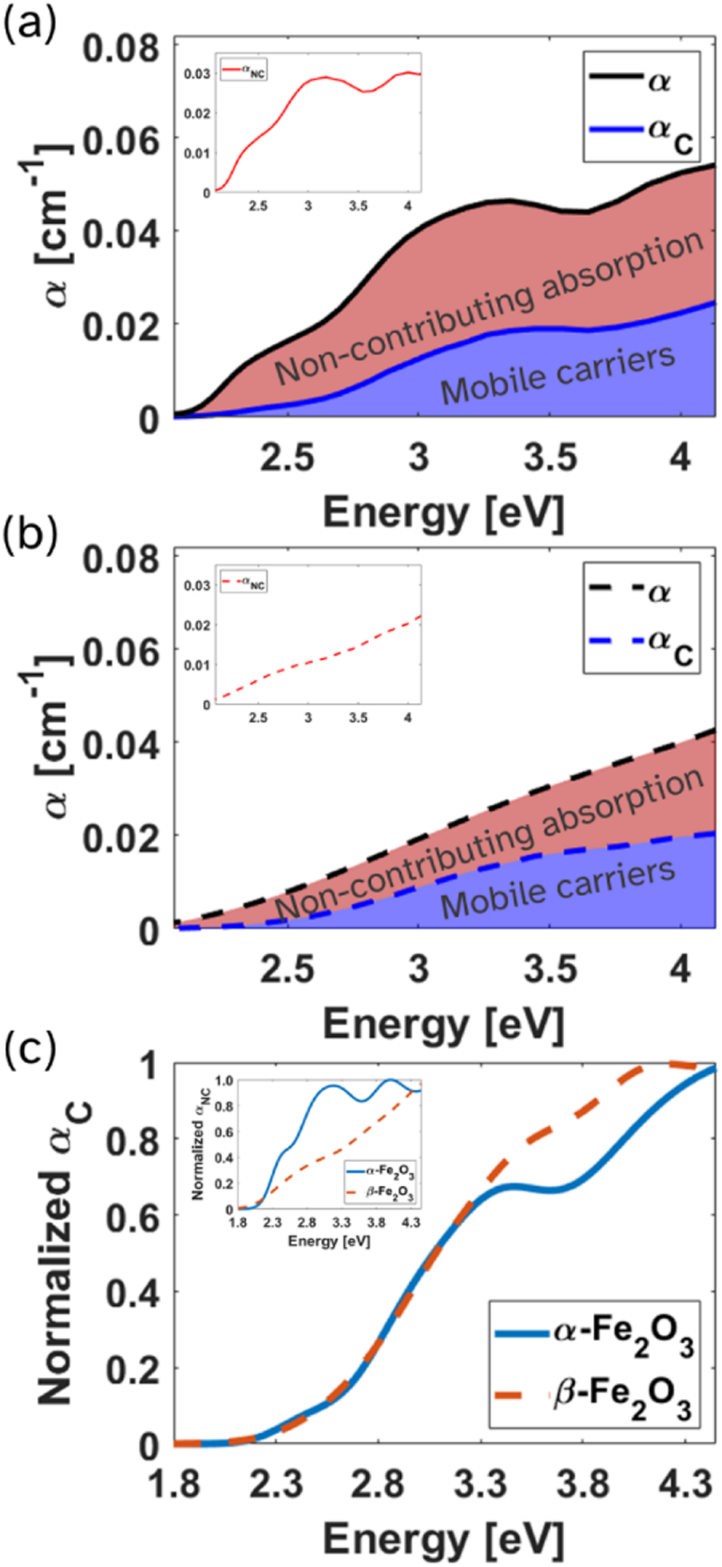
Absorption coefficient analysis. Total (black), contributing (blue) and noncontributing (red, inset) absorption coefficients of α-Fe_2_O_3_ (a, solid lines) and β-Fe_2_O_3_ (b, dashed lines). Shaded areas correspond to the fraction of contributing and noncontributing optical excitations out of the absorption coefficients. (c) α̃_C_ of α-Fe_2_O_3_ (blue, solid) and β-Fe_2_O_3_ (orange, dashed), showing that for both polymorphs the curves overlap up to 3.3 eV, while α̃_NC_ (inset) have differing profiles.

To compare the spectral behavior independent of absolute magnitude, both α_C_ and α_NC_ were normalized to their respective maxima, yielding the dimensionless spectra α̃_*C*_ and α̃_NC_ shown in Figure 5c. This normalization enables a direct comparison of the absorption mechanisms in the two polymorphs, regardless of their overall absorption intensity. The α̃_C_ spectra of both phases are nearly identical below 3.3 eV, despite their different crystal structures and coordination environments. Above 3.3 eV, deviations between the normalized spectra are visible, although interpretation in this region is complicated by increased parasitic absorption in the ITO layer. The similarity in the α̃_C_ spectra throughout most of the visible spectrum suggests that the optical transitions responsible for generating mobile charge carriers are not significantly affected by the difference in the crystal structures, implying they arise from a process governed by the local Fe–O bonding environment. Since ligand-to-metal charge-transfer (LMCT, O 2p → Fe 3d) transitions are known to generate mobile charge carriers in hematite, our results suggest LMCT excitation energies, and thus the onset of productive absorption does not shift appreciably between the two polymorphs.

In contrast, the α̃_NC_ spectra differ markedly between the two polymorphs. Hematite exhibits pronounced spectral features consistent with localized transitions previously assigned to LF excitations at energies of 2.3, 2.4, 2.9, and 3.6 ± 0.1 eV.^11,26,47,48^ On the other hand, the α̃_NC_ of β-Fe_2_O_3_ is relatively featureless across the absorption spectrum. Although β-Fe_2_O_3_ possesses a narrower bandgap (∼1.8 eV), this apparent advantage is largely optical rather than electronic as the red-shifted absorption edge corresponds mainly to noncontributing transitions, yielding little increase in the productive absorption α_C_. Thus, the additional long-wavelength absorption in β-Fe_2_O_3_ enhances total absorptance but not mobile-carrier generation.

The combined analysis of α̃_C_ and α̃_NC_ suggests that both polymorphs share the same LMCT-driven pathway for mobile charge carrier generation, while the α̃_NC_ spectra likely reflect differences in the underlying LF state manifold. Ultrafast relaxation into LF states provides a possible explanation for the subunity mobile charge carrier yields observed here, as carrier relaxation in hematite has been suggested to occur either through direct LF excitation of Fe^3+^ (3d^5^) centers or through rapid deactivation of carriers into LF states following LMCT excitation.^9,11^ Further investigation of the optical transitions and charge carrier dynamics in the β-Fe_2_O_3_ polymorph, which remain largely unexplored compared to hematite, will help clarify the specific relaxation pathways responsible for these losses. Nevertheless, the similarity in the α̃_C_ spectra found here suggests that modifying the long-range crystalline order, from α- to β-Fe_2_O_3_, does not significantly affect this loss pathway. Instead, it appears to be predominantly influenced by the local Fe–O electronic structure associated with the octahedrally coordinated Fe^3+^ (3d^5^) centers. Consequently, ξ_(λ)_ in iron oxides appears intrinsically limited by the local electronic structure, emphasizing the need for strategies that suppress LF-mediated relaxation.

## Conclusion

In summary, this work presents the first direct extraction of the spatial charge collection efficiency and mobile charge carrier photogeneration yield for β-Fe_2_O_3_ and compares these quantities with those of α-Fe_2_O_3_, using epitaxial thin-film photoanodes as model systems. While the α-Fe_2_O_3_ films exhibit larger hole transport lengths and correspondingly higher spatial charge collection efficiency, the spectral profile of the absorption that contributes to mobile charge-carrier generation closely overlaps for both polymorphs across the visible spectrum. This result establishes that the spectral dependence of contributing absorption in these epitaxial films is largely insensitive to polymorph-dependent structural variations and is instead governed primarily by the local electronic structure associated with octahedrally coordinated Fe^3+^ (3d^5^) centers.

The SCE analysis introduced here is well suited to flat, homogeneous thin-film photoelectrodes with minimal optical scattering, where carrier transport can be described within a simple, single-junction geometry. Extending this approach to more complex photoelectrode architectures, such as multilayer homojunction or heterojunction systems, represents an important future direction and will require the development of new SCE analysis methodologies, for example through regularization-based inversion of eq 1

More broadly, these results highlight that improving the performance of open d-shell transition-metal-oxide photoelectrodes will ultimately require strategies that directly enhance mobile charge carrier generation by suppressing nonproductive relaxation pathways associated with ligand-field states. While improvements in charge-carrier transport can increase charge collection, the invariance of the mobile carrier generation spectral profile across the polymorphs indicates that carrier generation constitutes a fundamental performance bottleneck that cannot be overcome through structural modification alone. The methodology and insights presented here provide a general framework for distinguishing carrier-generation-limited from transport-limited regimes in oxide photoelectrode materials, offering a pathway to improved device performance.

## Supplementary Material



## Notes

HRXRD and AFM measurements; detailed ellipsometry analysis; IMPS and Mott–Schottky measurements; thickness effect analysis; non-normalized absorption coefficient comparison; stability assessment of β-Fe_2_O_3_ through CV, chronoamperometry, HRXRD, and XPS measurements (PDF)
