# Supplementary material for: Disentangling Structural and Electronic Contributions to Photogenerated Mobile Charge Carrier Yield and Transport in Fe2O3 Polymorphs
Source: ACS Appl Mater Interfaces. 2026 Feb 19;18(8):12693–701. doi: 10.1021/acsami.5c23252 (PMC12964333; doi:10.1021/acsami.5c23252)
Supplement: Supplementary file 1 [file am5c23252_si_001.pdf]

Supporting Information

Disentangling Structural and Electronic Contributions to Photogenerated Mobile Charge Carrier Yield and Transport in Fe_2O_3 Polymorphs

Sa'ar Shor Peled [1,2], *Kumaraswamy Miriyala* [1,2] *and Daniel A. Grave** [1,2]

[1]Department of Material Engineering, Ben-Gurion University of the Negev, Beer Sheva 8410500, Israel

[2]Ilse Katz Institute for Nanoscale Science and Technology, Ben-Gurion University of the Negev, Beer Sheva 8410500, Israel

Email: dgrave@bgu.ac.il

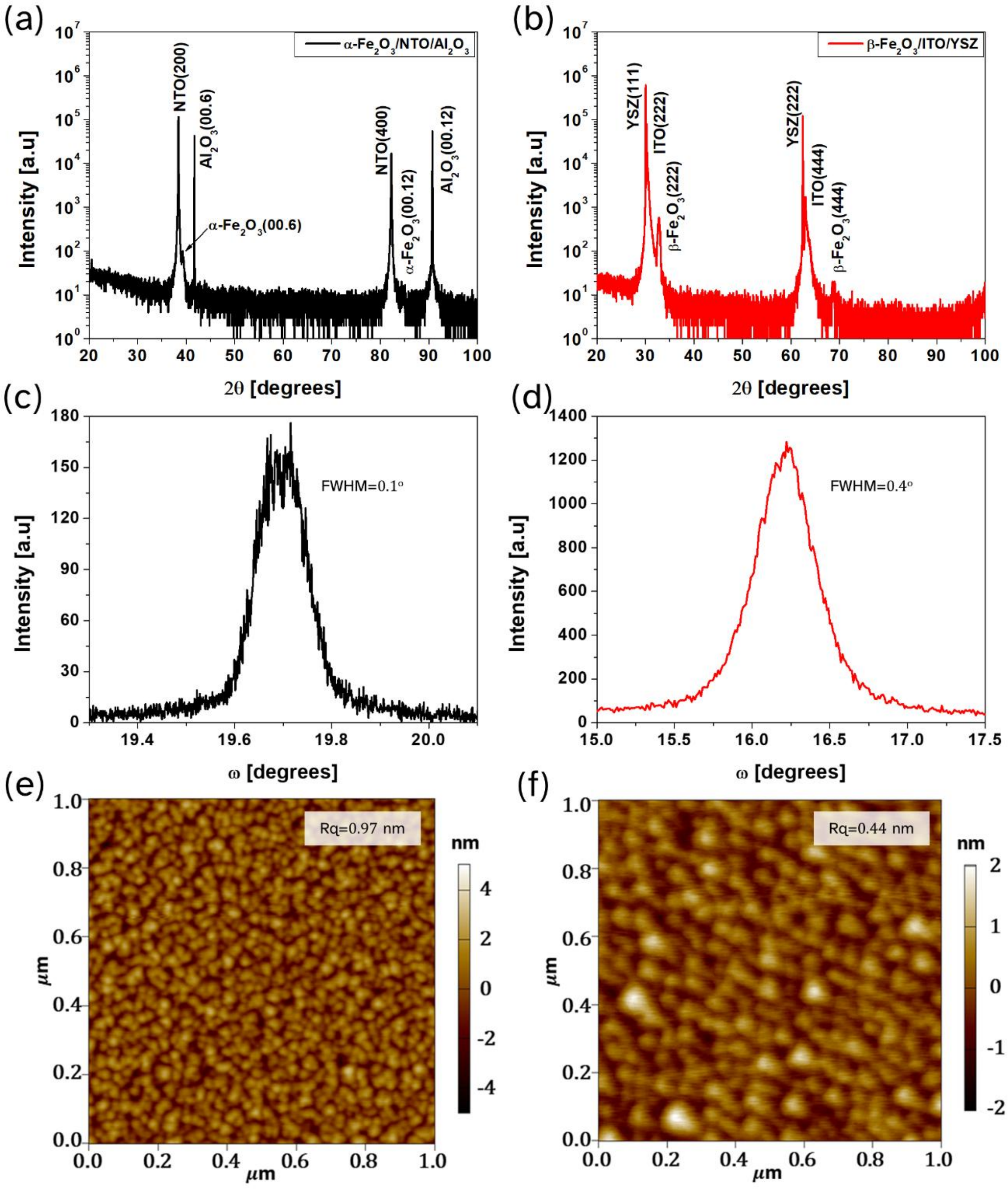

Figure S1- HRXRD θ-2θ patterns, rocking curves and AFM measurements of α-Fe_2O_3 (a, c and e) and β-Fe_2O_3 (b, d and f), showing phase-pure, highly oriented and smooth films.

Spectroscopic Ellipsometry Analysis:

To conduct the spatial collection analysis described in the main text, first the optical generation efficiency (OG) of the photoactive layer needs to be calculated. To that end, the complex refractive index of each layer was extracted via spectroscopic ellipsometry (SE) and was used to determine each layer's absorptance using Transfer-Matrix method simulations. First, bare Al_2O_3 (00.6) and YSZ (111) substrates were measured and modeled (Figure S2a and Figure S*2*d), then TCO/substrate (b and e) and finally the full stack of Fe_2O_3/TCO/substrate (c and f). Optical constants of each additional layer are presented below its corresponding SE measurement. A general oscillator model was assigned for each layer, and parameter details are given in

Table S*1*. Since both substrates are transparent in the measured range, a single Lorentz oscillator, located within the out-of-range UV region, was sufficient. PSemi-M0 oscillator was assigned for the above bandgap absorbance modes, while two Gaussians were used for ITO. For both TCOs, Drude oscillator was assigned to represent the free electron model. Iron oxides were well described using a single Tauc-Lorentz for the band edge absorbance mode, and several Gaussians for the above bandgap modes. The oscillators parameters are presented as given by the J.A. Woollam CompleteEase software. The optical constants extracted from the spectroscopic ellipsometry were verified using transfer-matrix method simulations of the reflectance and transmittance spectra, as shown in the main text.

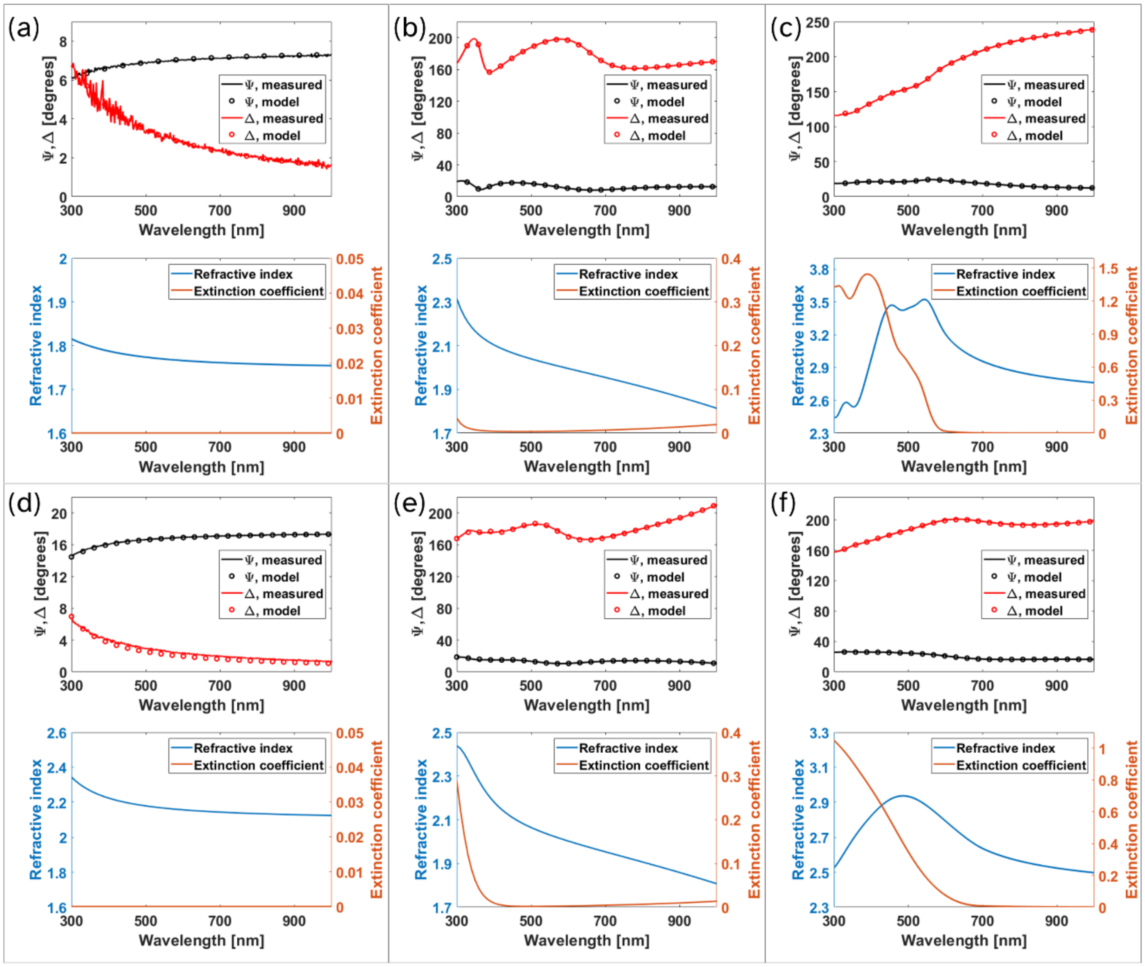

Figure S2- Spectroscopic ellipsometry modeling (top) and optical constants (bottom) of (a) Al_2O_3, (b) NTO, (c) α-Fe_2O_3, (d) YSZ, (e) ITO and (f) β-Fe_2O_3

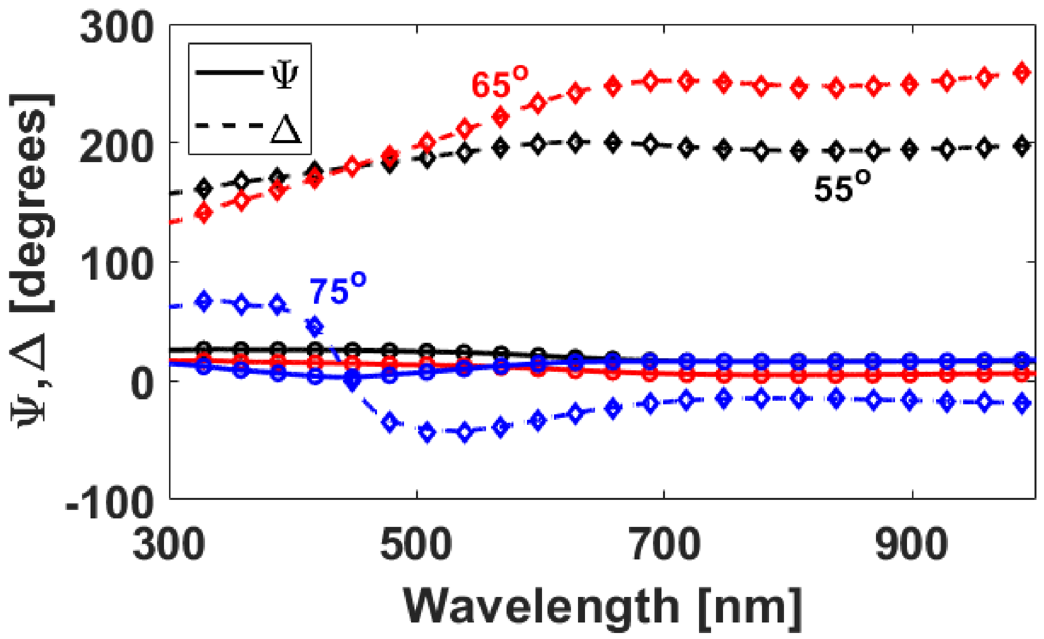

Figure S3- Spectroscopic ellipsometry modeling of β-Fe_2O_3 at 55, 65 and 75 ° angle of incidence (black, red and blue, respectively), where modelled spectra are marked with circles and diamonds for Ψ and Δ. Mean square error – 3.12.

Table S1- SE oscillators assignment

Layer	Oscillator	Oscillator Parameters
Al_2O_3	Lorentz	Amp=8.05<br>Br=1.6<br>E0=10.94
YSZ	Lorentz	Amp=22.77<br>Br=1.11<br>E0=7.37
NTO	Drude	N=1.42e20<br>μ=20.53<br>m*=0.26
	PSemi-M0	Amp=6.27<br>Br=0.56<br>E0=5.03<br>WR=1.98<br>PR=0.17<br>AR=0.41<br>O2R=-0.38
ITO	Drude	N=2.56e20<br>μ=64.281<br>m*=0.26
	Gaussian	Amp=2.13<br>Br=1.29<br>E0=4.65
	Gaussian	Amp=2.6<br>Br=1.64<br>E0=6.08

α-Fe_2O_3	Tauc-Lorentz	Amp=74.27<br>Br=0.34<br>E0=2.28<br>Eg=2.07
	Gaussian	Amp=0.49<br>Br=0.184<br>E0=2.52
	Gaussian	Amp=1.5<br>Br=0.38<br>E0=2.86
	Gaussian	Amp=7.14<br>Br=0.89<br>E0=3.15
	Gaussian	Amp=4.3<br>Br=0.55<br>E0=3.89
	Gaussian	Amp=6.9<br>Br=0.67<br>E0=4.47
β-Fe_2O_3	Tauc-Lorentz	Amp=41.13<br>Br=1.94<br>E0=2.68<br>Eg=1.8
	Gaussian	Amp=3.18<br>Br=1.86<br>E0=4.09
	Gaussian	Amp=2.97<br>Br=1.06<br>E0=5.04

Additional photoelectrochemical measurements:

IPCE measurement error

Figure S4 depicts the average value nad standard deviation of the IPCE measurements. Multiple IPCE measurements were performed, and the maximum deviation was calculated to be 1.16%.

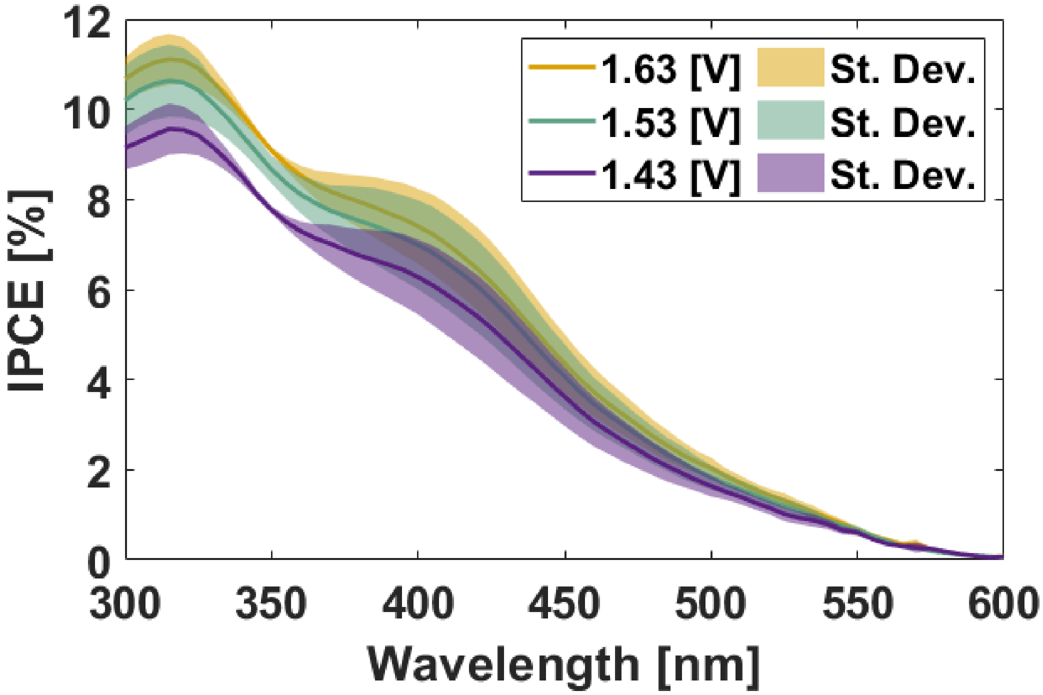

Figure S4- IPCE standard deviation calculation for 20 nm α-Fe_2O_3 film

Intensity-modulated photocurrent spectroscopy analysis

Determining the transfer efficiency η_t was carried out using IMPS measurements under 1000 W m^{-2} white LED irradiance, where the light intensity was modulated by 15% with frequencies ranging between 100 mHz and 10 kHz. Figure S5 shows the Nyquist plot of the IMPS measurement, with η_t calculated (as described in the main text) as the quotient of the low- and high-frequency intersect (LFI and HFI, respectively) with the real photocurrent axis (Y_{PC}‘).

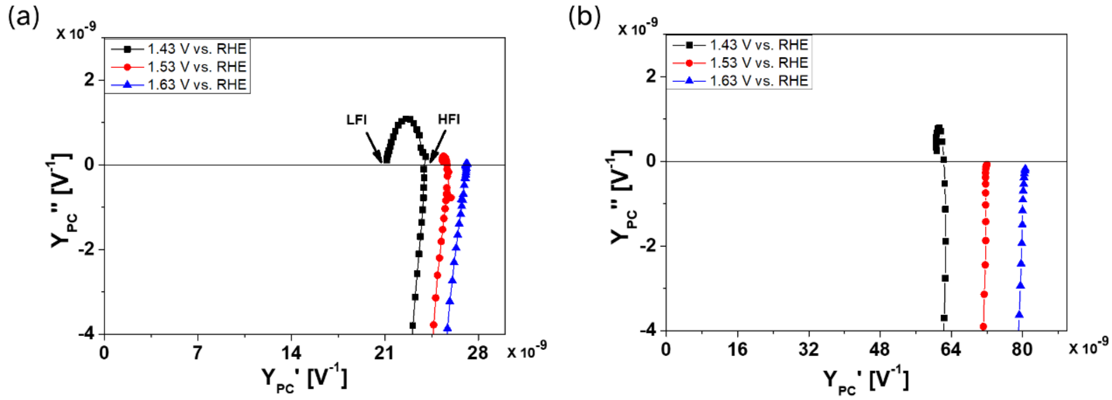

Figure S5- IMPS measurements of (a) α- and (b) β-Fe_2O_3 devices, measured at the electrical biases used for IPCE measurements. Examples of LFI and HFI of 1.43V measurement are marked in (a)

Mott-Schottky analysis

Mott-Schottky analysis was performed using Equation S1, and presented in Figure S6:

$$\frac{1}{C_{SC}^2} = \frac{2}{q\epsilon\epsilon_0 N_D}\left(V - V_{FB} - \frac{K_B T}{q}\right) \quad \text{(S1)}$$

Where C_{SC} is the capacitance of the space charge region, ϵ is the dielectric constant of iron oxide (considered as 33 [1]), ϵ_0 is vacuum permittivity (8.85e^{-14} F cm^{-1}), q is the elementary electric charge (1.6e^{-19} C), N_D is donor density, V is applied potential, V_{FB} is flat band potential, K_B is the Boltzmann constant (1.38e^{-23} J K^{-1}) and T is the absolute temperature. MS measurements were performed at frequencies of 5, 10 and 15 kHz, with Both polymorphs presented a linear region with a positive slope, consistent with an n-type photoanode. The depletion width was calculated according to Equation S2:

$$W = \sqrt{\frac{2\epsilon\epsilon_0(V - V_{FB})}{qN_D}} \quad \text{(S2)}$$

This calculation resulted in values exceeding the width of the films, therefore it was concluded that the films are fully depleted.

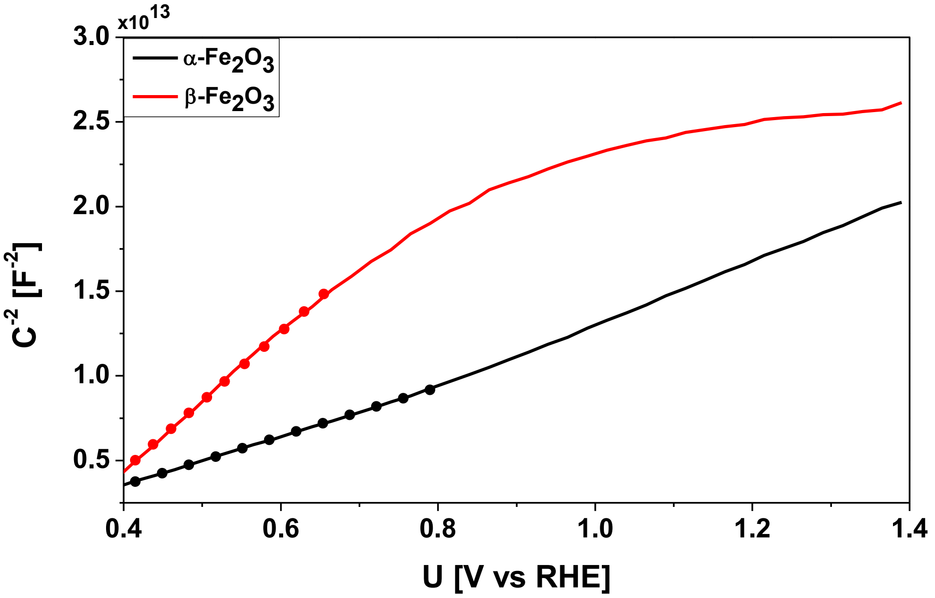

Figure S6- Mott-Schottky analysis of (black) α-Fe_2O_3 and (red) β-Fe_2O_3 measured at 10kHz, with linear region on which the analysis was performed is dotted

Analysis of thicker films:

Further verification of the analysis results was made by applying the decoupling process to thicker (28 nm) polymorph films, where the films are no longer fully depleted. Therefore, the SCE profile was fitted with the full piecewise equation presented in the main text (Equation 2). Figure S7a presents the calculated φ(x) profiles, and verfies that the trend presented in the 20 nm film case is reproduced in these films as well, with α phase exhibiting higher transport lengths compared to β-Fe_2O_3. Calculated ξ(λ) spectra (Figure S7b) are in agreement with those extracted from the 20 nm film analysis, further strengthening the confidence in the results. As before, IPCE spectra were reconstructed and showed good agreement to the measured spectra. Values for the transfer efficiency, hole transport lengths, and depletion widths are given in Table S2.

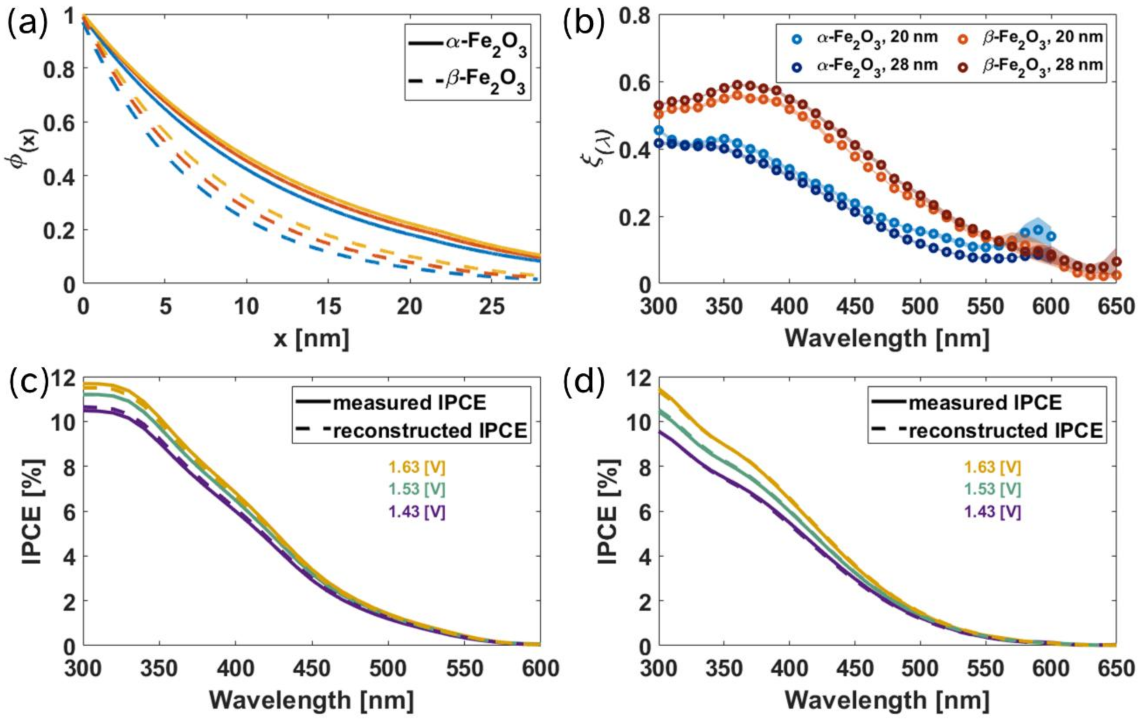

Figure S7- (a) $\phi_{(x)}$ profile of 28 nm α-Fe_2O_3 (solid) and β-Fe_2O_3 (dashed) under applied bias of 1.43, 1.53 and 1.63 V vs RHE (Blue, red and orange curves, respectively). (b) $\xi_{(\lambda)}$ spectra of 28 nm α-Fe_2O_3 (dark blue) and β-Fe_2O_3 (dark red), compared to PGY spectra of 20 nm films (light blue and light red for α- and β-Fe_2O_3, respectively). Measured and reconstructed IPCE spectra of 28 nm (c) α-Fe_2O_3 and (d) β-Fe_2O_3.

Table S2- transfer efficiency ($\phi_{(0)}$), drift length (L_{SCR}) depletion width (W) and diffusion length (L_{QNR}) of 20 and 28 nm polymorphs Errors in transport lengths were considered as the step size in the survey.

	**20 nm**							
	**α-Fe_2O_3**				**β-Fe_2O_3**			
**U vs RHE [V]**	$\boldsymbol{\phi_{(0)}}$	$\boldsymbol{L_{SCR}\,[nm]}$	$\boldsymbol{W\,[nm]}$	$\boldsymbol{L_{QNR}\,[nm]}$	$\boldsymbol{\phi_{(0)}}$	$\boldsymbol{L_{SCR}\,[nm]}$	$\boldsymbol{W\,[nm]}$	$\boldsymbol{L_{QNR}\,[nm]}$
**1.43**	0.88	10.4±0.1			0.91	6.4±0.1		
**1.53**	0.99	11.3±0.1	Fully depleted	---	0.99	6.8±0.1	Fully depleted	---
**1.63**	1.00	11.8±0.1			1.00	7.6±0.1		
	**28 nm**							
	**α-Fe_2O_3**				**β-Fe_2O_3**			
**U vs RHE [V]**	$\boldsymbol{\phi_{(0)}}$	$\boldsymbol{L_{SCR}\,[nm]}$	$\boldsymbol{W\,[nm]}$	$\boldsymbol{L_{QNR}\,[nm]}$	$\boldsymbol{\phi_{(0)}}$	$\boldsymbol{L_{SCR}\,[nm]}$	$\boldsymbol{W\,[nm]}$	$\boldsymbol{L_{QNR}\,[nm]}$
**1.43**	0.99	12.2±0.1	21		0.97	7.1±0.1	20.4	
**1.53**	1.00	12.7±0.1	21.5	10.2±0.1	0.99	7.9±0.1	21.1	6±0.1
**1.63**	1.00	13.3±0.1	22		1.00	8.7±0.1	21.7	

Absorption coefficient comparison:

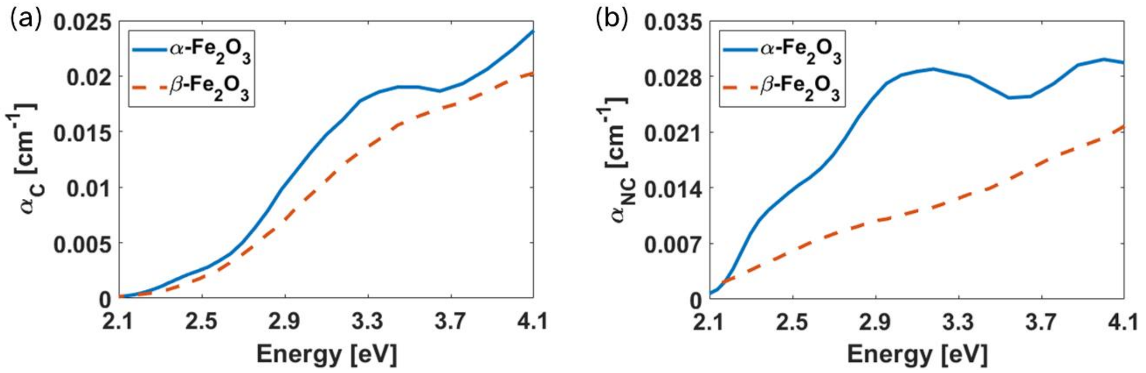

Figure S8- (a) Contributing and (b) non-contributing absorption coefficients comparison of α-Fe_2O_3 (blue, solid) and β-Fe_2O_3 (orange, dashed).

β-Fe_2O_3 stability:

Figure S9 summarizes the photoelectrochemical (PEC) performance and the structural and chemical stability of the epitaxial β-Fe_2O_3 film. Cyclic voltammetry and chronoamperometry measurements (Figure S9a and Figure S9b) demonstrate stable PEC performance during prolonged operation. High-resolution X-ray diffraction (HRXRD, Figure S9c) shows no detectable structural changes following long-term PEC testing, as evidenced by the Fe_2O_3 diffraction peak retaining its position, intensity, and full width at half maximum, clearly visible in the close-up of the Fe_2O_3 (222) peak presented in Figure S9d. The chemical stability of the film is assessed by X-ray photoelectron spectroscopy (XPS), shown in Figure S9e and Figure S9f. Comparison of the Fe 2p spectra acquired before and after long-term PEC operation reveals an exact overlap, indicating that the local chemical environment of iron remains unchanged. In addition, comparison between α- and β-Fe_2O_3 reference spectra highlights differences in line shape that are consistent with their deposition conditions (specifically, substrate temperature) rather than changes in oxidation state.[2] Detailed deconvolution of the Fe $2p_{3/2}$ region reveals a splitting of the feature near ~711 eV, which is consistent with intrinsic multiplet splitting of the Fe^{3+} $2p_{3/2}$ main line, arising from strong 2p–3d exchange interaction combined with spin-orbit and ligand-field splitting effects, predicted from experimental and ab-initio multiplet calculations.[3,4] This experimentally manifest as a resolved doublet or shoulder near ~711 eV even in the absence of any Fe^{2+} contribution. Deconvolution details are given in Table S3.

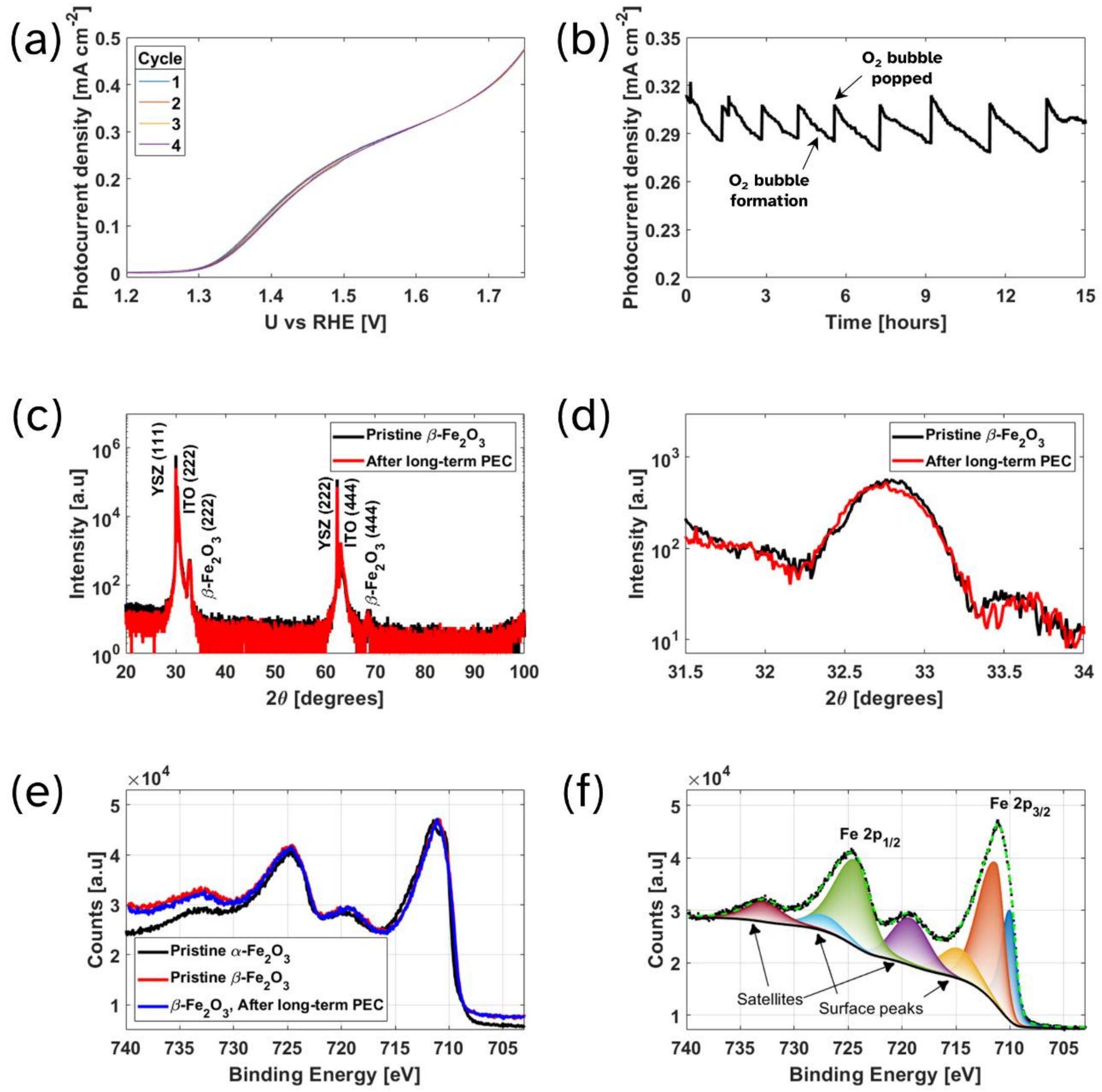

Figure S9- PEC, structural and chemical stability of β-Fe_2O_3. (a) cyclic voltammetry, performed under simulated 1 sun irradiance, (b) chronoamperometry measured under LED light bias at 1.53 V vs RHE, (c) high-resolution X-ray diffraction θ-2θ measurement before and after long-term PEC testing, (d) close-up of β-Fe_2O_3 (222) peak, (e) X-ray photoelectron spectra of pristine α-Fe_2O_3 (black), pristine β-Fe_2O_3 (red) and β-Fe_2O_3 after long-term PEC testing, and (f) β-Fe_2O_3 spectrum deconvolution.

Table S3- XPS spectrum deconvolution (Figure S9f) details

Peak ID	1	2	3	4	5	6	7
**Binding Energy [eV]**	709.995	711.275	714.732	719.243	724.308	727.2	732.8
**Counts [a.u]**	21000	27000	5710	8930	16400	2930	3810
**FWHM [eV]**	1.594	2.8	4	4	3.909	4.316	4

References:

(1) Piekner, Y.; Ellis, D. S.; Grave, D. A.; Tsyganok, A.; Rothschild, A. Wasted Photons: Photogeneration Yield and Charge Carrier Collection Efficiency of Hematite Photoanodes for Photoelectrochemical Water Splitting. *Energy Environ. Sci.* **2021**, *14* (8), 4584–4598.

(2) Lohaus, C.; Steinert, C.; Brötz, J.; Klein, A.; Jaegermann, W. Systematic Investigation of the Electronic Structure of Hematite Thin Films. *Adv. Mater. Interfaces* **2017**, *4* (20), 1700542.

(3) Bagus, P. S.; Nelin, C. J.; Brundle, C. R.; Crist, B. V.; Lahiri, N.; Rosso, K. M. Combined Multiplet Theory and Experiment for the Fe 2p and 3p XPS of FeO and Fe2O3. *J. Chem. Phys.* **2021**, *154* (9).

(4) Bagus, P. S.; Nelin, C. J.; Brundle, C. R.; Crist, B. V.; Lahiri, N.; Rosso, K. M. Origin of the Complex Main and Satellite Features in Fe 2p XPS of Fe 2 O 3. *Phys. Chem. Chem. Phys.* **2022**, *24* (7), 4562–4575.
